# The effects of purslane consumption on blood pressure, body weight, body mass index, and waist circumference: a systematic review and meta-analysis of randomised controlled

**DOI:** 10.1017/jns.2023.115

**Published:** 2023-12-27

**Authors:** Behnaz Narimani, Mohammad Reza Amini, Fatemeh Sheikhhossein, Camellia Akhgarjand, Mohammad Gholizadeh, Moein Askarpour, Azita Hekmatdoost

**Affiliations:** 1 Student Research Committee, Department of Clinical Nutrition and Dietetics, Faculty of Nutrition Sciences and Food Technology, National Nutrition & Food Technology Research Institute, Shahid Beheshti University of Medical Sciences, Tehran, Iran; 2 Department of Clinical Nutrition, School of Nutritional Sciences and Dietetics, Tehran University of Medical Sciences (TUMS), Tehran, Iran; 3 Department of Clinical Nutrition & Dietetics, National Nutrition & Food Technology Research Institute, Shahid Beheshti University of Medical Sciences, Tehran, Iran; 4 Student Research Committee, Department of Clinical Nutrition, School of Nutrition and Food Sciences, Shiraz University of Medical Sciences, Shiraz, Iran

**Keywords:** Blood pressure, Body weight, Meta-analysis, Purslane

## Abstract

The effects of purslane consumption on anthropometric measurements and blood pressure have been studied in numerous experiments. However, the research findings conflict with one another. In order to assess the impact of purslane on weight, body mass index (BMI), waist circumference (WC), systolic blood pressure (SBP), and diastolic blood pressure (DBP), this meta-analysis was carried out. Up until February 2023, PubMed, Web of Science, Scopus, Google Scholar, and the reference lists of the identified pertinent randomised controlled trials (RCTs) were all searched. The random-effects model was used to calculate the effect size and then to describe it as a weighted mean difference (WMD) and 95 % confidence interval (CI) (CRD42023427955). The systematic review was able to incorporate seven RCTs. Meta-analysis showed that purslane significantly decreased body weight (WMD): −0⋅73 kg, 95 % confidence interval (CI): −1⋅37, −0⋅09, *P*=0⋅025), BMI (WMD: −0⋅35 kg/m^2^, 95 % CI: −0⋅64, −0⋅07, *P*=0⋅016), and SBP (WMD: −3⋅64 mmHg, 95 % CI: −6⋅42, −0⋅87, *P* = 0⋅01), and for WC, there was no discernible effect (WMD: −0⋅86 cm; 95 % CI, −1⋅80 to 0⋅07; *P* = 0⋅06) and DBP (WMD: −0⋅36 mmHg; 95 % CI, −1⋅75 to 1⋅03; *P* = 0⋅61). Purslane consumption, especially in participants with a BMI of <30, might play a role in decreasing SBP, body weight, BMI, and WC. Purslane consumption significantly reduced body weight, BMI, and SBP; however, WC and DBP did not experience a reduction. More investigation is needed to verify the impact of purslane consumption on anthropometric parameters and blood pressure.

## Introduction

Obesity and overweight, which are frequently recognised as substantial health risks on a global scale, have a considerable impact on a number of non-communicable diseases, including diabetes, cardiovascular disease (CVD), and cancer.^(1,2)^ More than 1⋅9 billion adults over the age of 18 years were found to be overweight in a World Health Organisation (WHO) report.^(1)^ This number continues to rise. By 2030, according to estimates, more than 2⋅16 billion adults and children will become less healthy as a result of being overweight or obese.^(3)^ Exercise, nutrition therapy, behavioural interventions, prescription drugs, and surgical procedures are common methods of treating obesity.^(4,5)^ To manage obesity, anti-obesity supplements are frequently employed. Medicinal plants have been used for a long time to control obesity and are linked to better and faster weight loss.^(6,7)^ Although there are many different weight-management supplements on the market, it is still unclear how effective the majority of them are. One of the most significant medicinal plants is purslane. Omega-3 fatty acids, beta-carotene,^(8)^ tocopherol, glutathione,^(9)^ amino acids, ascorbic acid, and phenolic compounds^(10)^ are only a few of the biologically active ingredients found in them. The bioactive components of purslane are flavonoids, which have biologically pharmacological properties including anti-microbiological, antiviral, antioxidant, and anti-inflammatory properties. The highest levels of flavonoids are observed in roots, stems, and leaves, respectively. The studies reported seven different flavonoids, such as myricetin, kaempferol, apigenin, luteolin, genistein, quercetin, and genistin.^(11–14)^

According to WHO data, purslane is the most commonly used medicinal plant. The name ‘Global Panacea’ refers to a comprehensive elixir or panacea.^(15)^ This plant boosts immunity and fights diabetes, and bacterial, and viral infections. As a result, it is regarded as a long-lived plant in the Chinese literature.^(16–18)^ Purslane plays an important role in mice with diabetic conditions by preventing diabetic vascular inflammation, endothelial dysfunction, and hyperglycaemia. In addition, in alloxan-induced diabetic mice, purslane modulates glucose and lipid metabolism by lowering the glucose level.^(19–21)^

Clinical research that looked at how purslane supplementation affected anthropometric measurements and blood pressure produced mixed results. The impact of this supplement on the aforementioned parameter was said to have improved according to a number of research.^(22–26)^ However, additional research failed to support the effect.^(27,28)^ The precise impact of purslane on weight is still unclear and requires clarification. It should be noted that blood pressure and anthropometric measurements may not have been specifically included in the titles or abstracts of the randomised controlled trials (RCTs), which may have evaluated these variables as their secondary outcome. To assess the impact of purslane supplementation on anthropometric measurements and blood pressure, a highly sensitive search technique was used in the current systematic review and meta-analysis to include the most RCTs possible.

## Method

This systematic review and meta-analysis were conducted and reported using the PRISMA (Preferred Reporting Items for Systematic Review and Meta-analysis) protocol (Supplementary Table 1). The study's protocol has been registered in the PROSPERO (CRD42023427955).

### Search strategy

We searched PubMed/Medline, Web of Science, Scopus, and EMBASE systematically up to February 2023 in search of papers. We used a combination of MeSH and non-MeSH terms, including (Portulaca OR ‘Portulaca oleracea’ and Purslane) AND (intervention OR random OR RCT OR randomised OR Placebo OR Randomly OR Assignment OR trial OR randomised OR Cross-Over OR ‘Double-Blind’). There was no time limit, and we included only English-language articles. We further checked the references of the articles and reviews that were included in order to confirm that our literature search was thorough, and we conducted a meta-analysis of additional pertinent studies.

### Inclusion criteria

After searching in databases, the studies were imported into Endnote version X9. After that, one of the writers checked the included studies’ titles and abstracts (B.N.). Potentially related full-text papers were examined by two separate investigators (C.A. and M.R.A.), and any disagreements were settled in conjunction with the chief reviewer. The inclusion of articles was determined by the following standards: (1) trials were performed on adults (age over 18 years), (2) studies that examined the impact of a purslane intervention on anthropometric parameters and blood pressure compared to a placebo; and (3) RCTs with a cross-over or parallel configuration.

### Exclusion criteria

If a study matched one or more of the following requirements, it was ignored: (1) They involved animals, children, pregnant or lactating women; or (2) they used a non-RCT design, such as letters, conference papers, protocol studies, or observational research; (3) studies with no placebo group; (4) purslane alone was unable to determine the effects, so they were investigated in combination with other substances.

### Screening and data extraction

The information below was gleaned from the included trials by two impartial reviewers (C.A. and M.R.A.): the number of people in the placebo and intervention groups, the mean age and sex of the subjects, the author's first name, the subjects’ health status, the year of publication, the length of supplementation, the dosage and type of purslane supplement, the study location, and the mean standard deviation (sd), systolic blood pressure (SBP), diastolic blood pressure (DBP), body mass index (BMI), weight, and waist circumference (WC) values prior to and following supplementation in both intervention and controls were entered into a standardised spreadsheet at the start of the study and following the intervention in each group. For trials reporting effects at different time points, we considered end-of-study information. By dividing the sample size by the number of doses, studies exploring the effects of various doses were evaluated, with each dose being evaluated as independent research.

### Risk-of-bias assessment

Using the Cochrane Risk-of-Bias tool for clinical trials, the included RCTs’ potential for bias was evaluated. Two independent researchers (C.A. and M.R.A.) assessed each publication's quality using the following criteria: (a) selective reporting, (b) blinding of participants and personnel, (c) completeness of study outcome information, (d) allocation concealment, (e) random sequence generation, (f) other possible sources of biases, and (g) blinding of study outcome examination. A bias label (low risk, high risk, or unknown risk of bias) was given to each study (Table 1).

**Table 1.** Risk of bias for randomised controlled trials, assessed according to the Revised Cochrane risk-of-bias tool for randomised trials (RoB 2)

Publications	Random sequence generation	Allocation concealment	Selective reporting	Blinding (participants and personnel)	Blinding (outcome assessment)	Incomplete outcome data	other source of bias
1. Adelnia Najafabadi (2016)	L	U	L	H	H	L	L
2. Darvish Damavandi (2020)	L	U	L	L	U	H	L
3. Esmaillzadeh (2015)	L	U	L	H	H	L	L
4. Gheflati (2019)	L	U	L	H	H	L	L
5. Papoli (2019)	L	U	L	H	H	L	L
6. Wainstein (2016)	L	U	L	L	U	H	L
7. Ghorbanian (2019)	L	U	L	U	U	L	L

L, low risk of bias; H, high risk of bias; U, unclear risk of bias.

### Data synthesis and statistical analysis

Here is a meta-analysis. Based on the mean changes and SDs of SBP, DBP, weight, BMI, and WC, DerSimonian, and Laird random-effect models^(29)^ were used to calculate pooled effect sizes. We calculated within-group mean differences by subtracting the final mean from each group's baseline mean value. In order to get the SDs of the mean differences, the following formula was used: sd change = square root [(sd baseline)^2^ + (sd final)^2^ – (2 × 0⋅8 × sd baseline × sd final)] if they were not reported.^(30)^

To calculate sd the following formula was utilised when the trials reported the standard error of the mean (S.E.M.): S.D ¼ S.E.M. *n* (*n* is the number of subjects in each group). Eventually, two parameters: weighted mean difference (WMD) and 95 % CI, reported the magnitude of the overall effect size in a random effect model. We applied *I*-square (*I*^2^) test, with a significance level of *P* < 0⋅10 to determine heterogeneity among the results of included trials.^(31,32)^ In order to determine the most likely reason for heterogeneity, subgroup analyses depending on the trial's duration, the mean age, the sex of the participants, the individuals’ BMI, and the kind of intervention were carried out. Overall effect size robustness was analyzed using the one-point exclusion method, which excluded each study individually and repeated the analysis.^(32)^ Additionally, to identify publication bias, Egger's regression test and a visual inspection of the funnel plot were used.^(33)^ All statistical analyses were performed using Stata Version 14.0 (Stata Corp, College Station, TX, USA), with a statistical significance cutoff of *P* < 0⋅05.

## Results

### Study selection

**Fig. 1.**
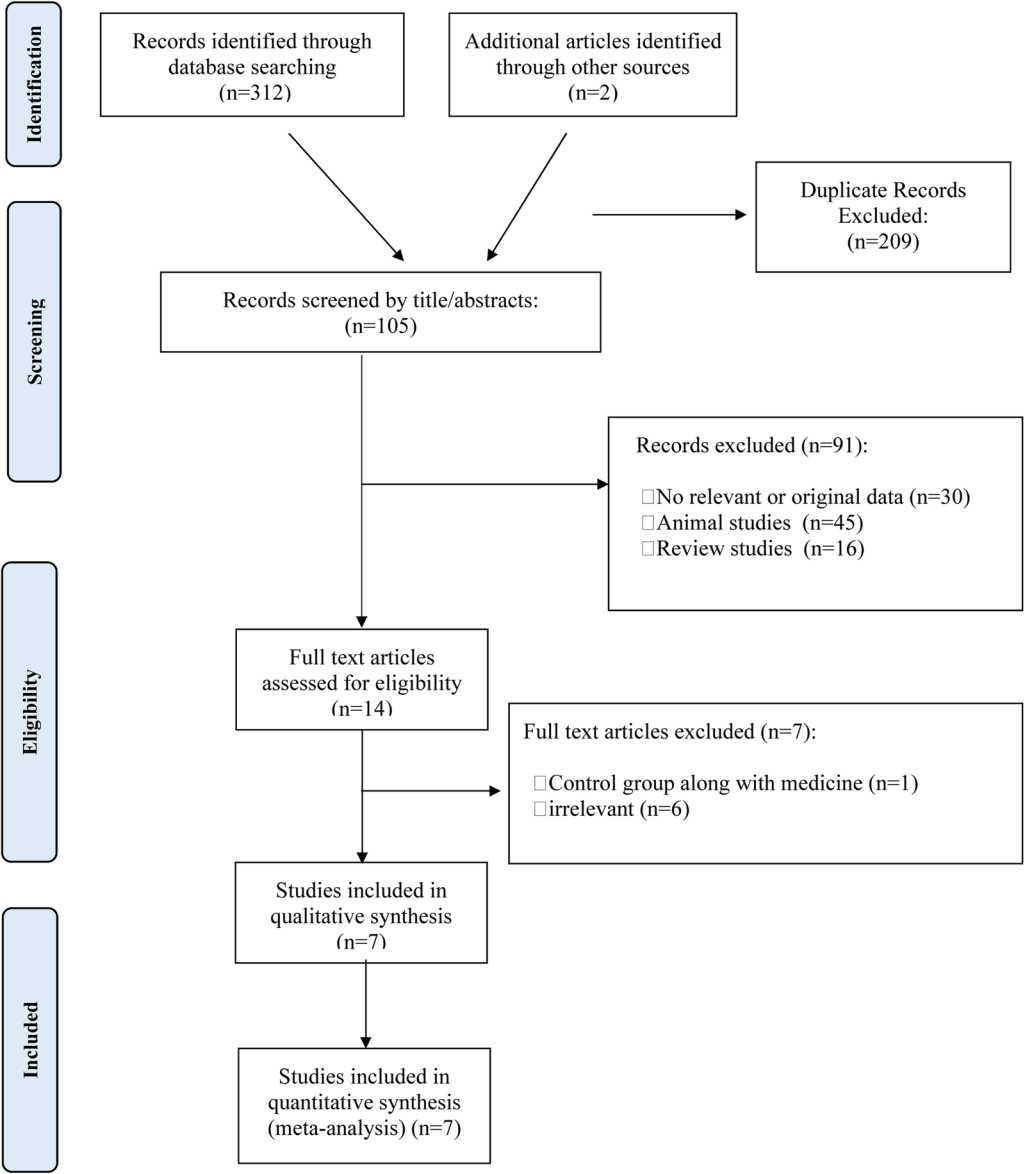
Flow chart of the number of studies identified and selected into the meta-analysis.

In the initial search, 314 articles were found in total. 209 duplicate studies were removed from this list, and 105 papers were assessed based on their titles and abstracts and excluded. After that, fourteen studies were evaluated in full-text, and citations from those studies were eliminated for the following reasons: Control group along with medicine (*n* 1), irrelevant (*n* 6). Finally, the final quantitative analysis included seven trials.^(22–28)^ Fig. 1 depicts the flow chart of the comprehensive steps of the literature search procedure.

### Study characteristic

The detailed characteristics of the seven trials are shown in Table 2. Overall, 360 subjects participated in these trials. Trials were published between 2015 and 2020. The intervention duration varied from 5 weeks to 12 weeks, and the mean age of the subjects ranged from 25⋅4 to 55⋅3 years. All the trials were RCTs. Two studies were exclusively performed on females,^(24,25)^ and all others were performed on both sexes. Among these trials, subjects were from different populations, including metabolic syndrome (MetS),^(25)^ healthy adults,^(24)^ participants with nonalcoholic fatty liver disease (NAFLD),^(22,27,28)^ and type 2 diabetes mellitus (T2DM).^(23–26)^

**Table 2.** Demographic characteristics of the included studies

First author (year)	Location	Study design	Health status	Sex	Sample size	Duration (week)	Mean age (year)	Baseline BMI (kg/m^2^)	Intervention group	Comparator group	Outcome
1. Esmaillzadeh *et al.* (2015)	Iran	RCT	T2DM	Both	47	5	51⋅4	28⋅9	10 g/day purslane seeds with 240 cc low-fat yogurt	240 cc low-fat yogurt	SPB/DBP/Weight/BMI/WC
2. Adelnia Najafabadi *et al.* (2016)	Iran	RCT	NAFLD	Both	54	8	39⋅9	31⋅9	10 g/day purslane seeds and a low-calorie diet	A low-calorie diet	Weight/BMI/WC
3. Wainstein *et al.* (2016)	Israel	RCT	T2DM	Both	50	12	55⋅3	29⋅5	180 mg purslane extract	Placebo	SPB/DBP/Weight/BMI
4. Papoli *et al.* (2019)	Iran	RCT	Metabolic syndrome	Female	64	12	43⋅6	27⋅2	10 g/day purslane seeds with 240 cc low-fat yogurt	240 cc low-fat yogurt	SPB/DBP/Weight/BMI/WC
5. Ghorbanian *et al.* (2019)	Iran	RCT	Healthy	Female	20	8	25⋅4	27⋅6	1200 mg portulaca supplementation	Placebo	Weight/BMI
6. Gheflati *et al.* (2019)	Iran	RCT	NAFLD	Both	54	8	39⋅9	31⋅9	10 g/day purslane seeds and a low-calorie diet	A low-calorie diet	SPB/DBP
7. Darvish Damavandi *et al.* (2020)	Iran	RCT	NAFLD	Both	71	12	46⋅1	31⋅7	300 mg purslane extract	Placebo	SPB/DBP/Weight/BMI/WC

RCT, randomised controlled trial; SBP, systolic blood pressure; DBP, diastolic blood pressure; BMI, body mass index; WC, waist circumference; T2DM, type 2 diabetes mellitus; NAFLD, nonalcoholic fatty liver disease.

### Effect of purslane on SBP

**Fig. 2.**
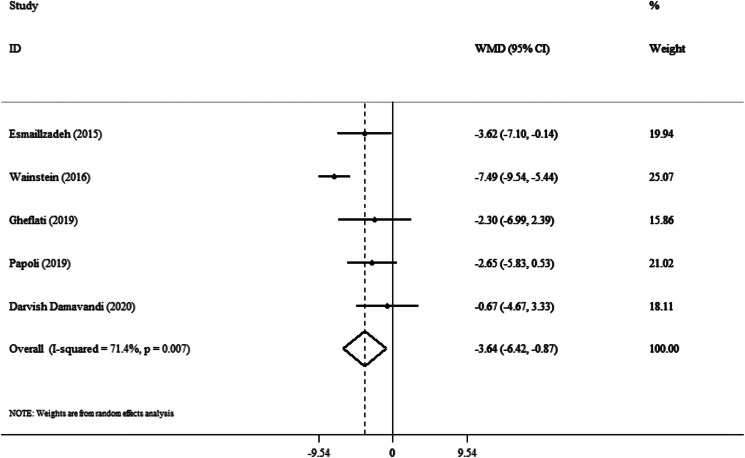
Forest plot detailing weighted mean difference and 95 % confidence intervals (CIs) for the effect of purslane on SBP.

The impact of the purslane intervention on SBP was examined in a total of five trials (including 286 people, including 142 in the intervention group and 144 in the control group). Based on the random-effects model, the purslane intervention had a significant effect on SBP (WMD: −3⋅64 mmHg; 95 % CI, −6⋅42 to −0⋅87; *P* = 0⋅01) (Fig. 2). In addition, substantial heterogeneity was identified between studies (I^2^ = 71⋅4 %; *P* = 0⋅007). Based on the subgroup analysis, a significant reduction in SBP was observed in trials where participants’ BMI in the range of 25–30 (WMD: −5⋅59; 95 % CI, −7⋅13 to −4⋅05; *P* < 0⋅001) (Table 3).

**Table 3.** Subgroup analysis of included randomised controlled trials in meta-analysis of the effect of purslane on blood pressure, weight, BMI, and WC

Group	No. of trials	WMD (95 % CI)	*P* value	*I^2^* (%)	*P*-heterogeneity	*P* for between subgroup heterogeneity
*SBP*						
Pooled effect size	5	−3⋅64 (−6⋅42, −0⋅87)	0⋅010	71⋅4	0⋅007	-
Duration (week)						0⋅205
≤8	2	−3⋅15 (−5⋅95, −0⋅36)	0⋅027	0⋅0	0⋅65	
>8	3	−5⋅23 (−6⋅81, −3⋅65)	<0⋅001	83⋅6	0⋅002	
Sex						0⋅156
Both	4	−5⋅20 (−6⋅73, −3⋅68)	<0⋅001	75	0⋅007	
Female	1	−2⋅65 (−5⋅83, 0⋅53)	0⋅102	–	–	
Mean age						0⋅290
≤40	1	−2⋅30 (−6⋅99, 2⋅39)	0⋅337	–	–	
>40	4	−4⋅95 (−6⋅39, −3⋅51)	<0⋅001	76⋅7	0⋅005	
Mean BMI						0⋅015
25–30	3	−5⋅59 (−7⋅13, −4⋅05)	<0⋅001	74⋅5	0⋅020	
≥30	2	−1⋅36 (−4⋅40, 1⋅69)	0⋅383	0⋅0	0⋅60	
Type of intervention						0⋅027
Seeds	3	−2⋅93 (−5⋅03, −0⋅83)	0⋅006	0⋅0	0⋅88	
Extract	2	−6⋅07 (−7⋅90, −4⋅25)	<0⋅001	88⋅7	0⋅003	
*Weight*						
Pooled effect size	6	−0⋅73 (−1⋅37, −0⋅09)	0⋅025	54⋅5	0⋅051	-
Duration (week)						0⋅163
≤8	3	−0⋅57 (−1⋅04, −0⋅10)	0⋅017	73	0⋅025	
>8	3	−1⋅06 (−1⋅58, −0⋅55)	<0⋅001	0⋅0	0⋅440	
Sex						0⋅030
Both	4	−0⋅46 (−0⋅92, −0⋅00)	0⋅049	34⋅6	0⋅205	
Female	2	−1⋅23 (−1⋅76, −0⋅71)	<0⋅001	41⋅4	0⋅191	
Mean age						0⋅256
≤40	2	−0⋅27 (−1⋅23, 0⋅69)	0⋅578	85⋅6	0⋅008	
>40	4	−0⋅87 (−1⋅24, −0⋅50)	<0⋅001	0⋅0	0⋅428	
Mean BMI						0⋅061
25–30	4	−0⋅93 (−1⋅30, −0⋅55)	<0⋅001	43⋅8	0⋅148	
≥30	2	0⋅01 (−0⋅90, 0⋅91)	0⋅987	53⋅3	0⋅143	
Type of intervention						0⋅214
Seeds	3	−0⋅74 (−1⋅11, −0⋅38)	<0⋅001	70⋅3	0⋅0⋅35	
Extract	2	−0⋅60 (−2⋅03, 0⋅82)	0⋅407	28⋅0	0⋅276	
Supplementation	1	−2⋅43 (−4⋅30, −0⋅56)	0⋅011	–	–	
*BMI*						
Pooled effect size	6	−0⋅35 (−0⋅64, −0⋅07)	0⋅016	62	0⋅022	-
Duration (week)						0⋅120
≤8	3	−0⋅25 (−0⋅45, −0⋅06)	0⋅011	80⋅7	0⋅006	
>8	3	−0⋅46 (−0⋅63, −0⋅28)	<0⋅001	0⋅0	0⋅832	
Sex						0⋅031
Both	4	−0⋅23 (−0⋅41, −0⋅05)	0⋅014	5⋅6	0⋅365	
Female	2	−0⋅51 (−0⋅70, −0⋅33)	<0⋅001	81⋅3	0⋅021	
Mean age						0⋅772
≤40	2	−0⋅27 (−0⋅96, 0⋅43)	0⋅458	90⋅4	0⋅001	
>40	4	−0⋅37 (−0⋅50, −0⋅24)	<0⋅001	0⋅0	0⋅441	
Mean BMI						0⋅216
25–30	4	−0⋅39 (−0⋅53, −0⋅26)	<0⋅001	66⋅2	0⋅031	
≥30	2	−0⋅09 (−0⋅55, 0⋅37)	0⋅701	63⋅7	0⋅097	
Type of intervention						0⋅041
Seeds	3	−0⋅35 (−0⋅49, −0⋅22)	<0⋅001	70⋅4	0⋅0⋅34	
Extract	2	−0⋅33 (−0⋅78, 0⋅13)	0⋅158	0⋅0	0⋅937	
Supplementation	1	−1⋅97 (−3⋅22, −0⋅72)	0⋅002	–	–	
*WC*						
Pooled effect size	4	−0⋅83 (−1⋅80, 0⋅07)	0⋅069	65⋅1	0⋅035	-
Duration (week)						0⋅007
≤8	2	−0⋅29 (−0⋅74, 0⋅16)	0⋅211	0⋅0	0⋅795	
>8	2	−1⋅59 (−2⋅42, −0⋅76)	<0⋅001	14⋅9	0⋅278	
Sex						0⋅006
Both	3	−0⋅35 (−0⋅78, 0⋅07)	0⋅105	0⋅0	0⋅626	
Female	1	−1⋅93 (−2⋅97, −0⋅90)	<0⋅001	–	–	
Mean age						0⋅599
≤40	1	0⋅00 (−2⋅22, 2⋅22)	1⋅000	–	–	
>40	3	−0⋅60 (−1⋅01, −0⋅20)	0⋅003	76	0⋅016	
Mean BMI						0⋅831
25–30	2	−0⋅57 (−0⋅99, −0⋅15)	0⋅008	87⋅5	0⋅005	
≥30	2	−0⋅71 (−1⋅88, 0⋅47)	0⋅239	0⋅0	0⋅463	
Type of intervention						0⋅559
Seeds	3	−0⋅55 (−0⋅96, −0⋅14)	0⋅009	75⋅8	0⋅016	
Extract	1	−0⋅98 (−2⋅36, 0⋅40)	0⋅165	–	–	

SBP, systolic blood pressure; DBP, diastolic blood pressure; BMI, body mass index; WC, waist circumference.

### Effect of purslane on DBP

**Fig. 3.**
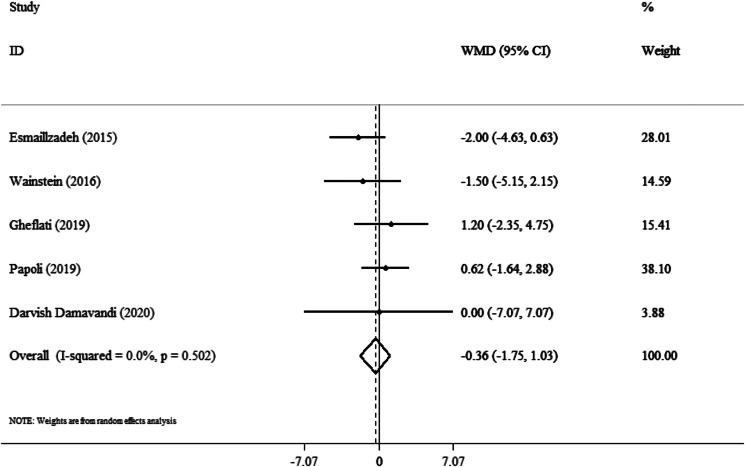
Forest plot detailing weighted mean difference and 95 % confidence intervals (CIs) for the effect of purslane on DBP.

The meta-analysis of five studies showed no significant change in DBP after purslane intervention (WMD: −0⋅36 mmHg; 95 % CI, −1⋅75 to 1⋅03; *P* = 0⋅61), without considerable study-to-study heterogeneity (*I*^2^ = 0⋅0 %; *P* = 0⋅502) (Fig. 3).

### Effect of purslane on weight

**Fig. 4.**
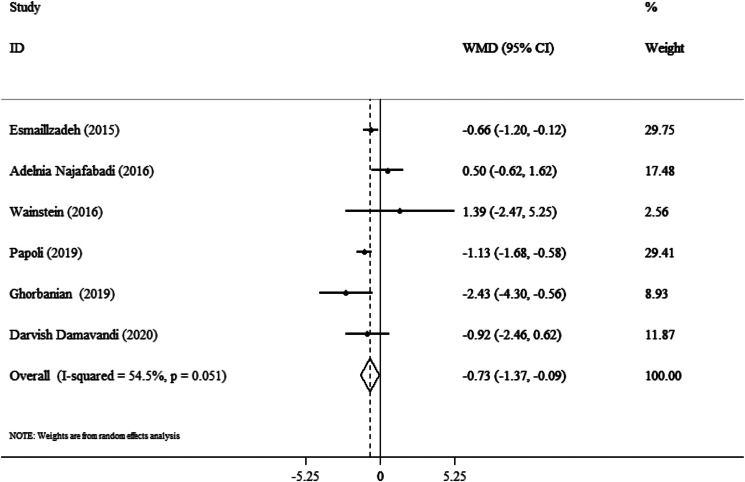
Forest plot detailing weighted mean difference and 95 % confidence intervals (CIs) for the effect of purslane on weight.

Six trials including 360 participants reported the effect of purslane intervention compared to placebo. The combination of effect sizes, obtained from the random-effects model, had a significant result in weight changes (WMD: −0⋅73 kg; 95 % CI, −1⋅37 to −0⋅09; *P* = 0⋅02) with a moderate heterogeneity between trials (I^2^ = 54⋅5 %; *P* = 0⋅05) (Fig. 4). Additionally, these findings indicated a significant loss of weight under the following circumstances: (1) age >40 years (WMD: −0⋅87 kg; 95 % CI, −1⋅24 to −0⋅50; *P* < 0⋅001), and (2) BMI in the range of 25–30 (WMD: −0⋅93 kg; 95 % CI, −1⋅30 to −0⋅55; *P* < 0⋅001) (Table 3).

### Effect of purslane on BMI

**Fig. 5.**
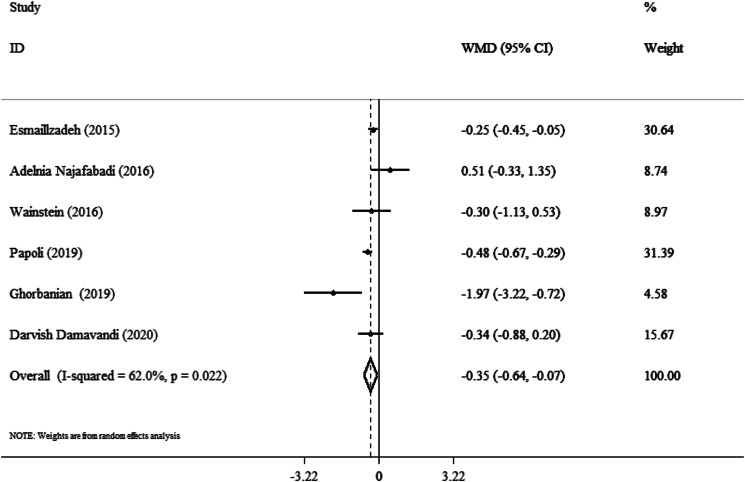
Forest plot detailing weighted mean difference and 95 % confidence intervals (CIs) for the effect of purslane on the BMI.

BMI change was considerably impacted by purslane intervention, according to results from six studies combined using the random-effects model (WMD: −0⋅35 kg/m^2^; 95 % CI, −0⋅64 to −0⋅07; *P* = 0⋅01) (Fig. 5). The studies showed a moderate heterogeneity level (*I*^2^ = 62⋅0 %, *P* = 0⋅02). The BMI of patients over 40 years old significantly decreased as a result of the purslane intervention (WMD: −0⋅37 kg/m^2^; 95 % CI, −0⋅50 to −0⋅24; *P* < 0⋅001), BMI in the range of 25–30 (WMD: −0⋅39 kg/m^2^; 95 % CI, −0⋅53 to −0⋅26; *P* < 0⋅001) (Table 3).

### Effect of purslane on WC

**Fig. 6.**
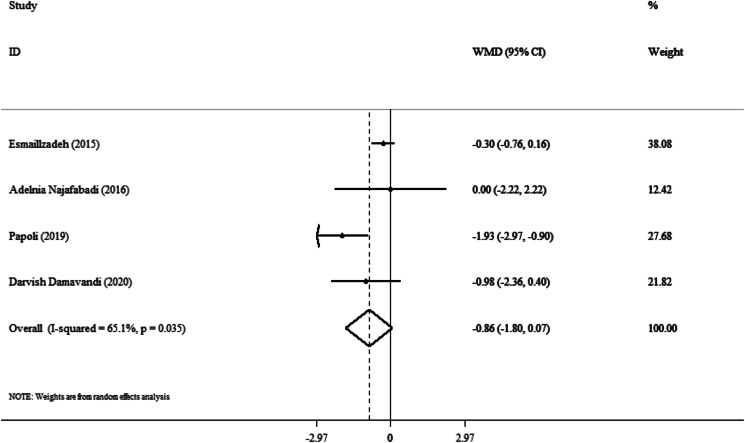
Forest plot detailing weighted mean difference and 95 % confidence intervals (CIs) for the effect of purslane on WC.

The impact of purslane intervention against placebo on the WC was assessed in four studies. Overall estimations showed no appreciable difference in WC between the intervention and control groups (WMD: −0⋅86 cm; 95 % CI, −1⋅80 to 0⋅07; *P* = 0⋅06) (Fig. 6) with a moderate degree of study heterogeneity (I^2^ = 65⋅1 %, *P* = 0⋅03). The purslane intervention caused a significant decrease in WC in trials that lasted more than 8 weeks (WMD: −1⋅59 cm; 95 % CI, −2⋅42 to −0⋅76; *P* < 0⋅001), and BMI < 30 (WMD: −0⋅57 cm; 95 % CI, −0⋅99 to −0⋅15; *P* = 0⋅008) (Table 3).

### Sensitivity analysis and publication bias

We performed the sensitivity analysis for SBP, DBP, weight, BMI, and WC to determine the influence of a single trial on the pooled effect sizes. The findings showed that, in the leave-one-out sensitivity analysis, the impact sizes were robust and that deleting none of the studies would modify them (Supplementary Figures 1–5). Begg's rank correlation examined the publication bias of the included studies. Begg's test results revealed no evidence of a publication bias for SBP (*P* = 0⋅327), DBP (*P* = 0⋅624), weight (*P* = 0⋅851), BMI (*P* = 0⋅851), and WC (*P* = 1⋅000).

## Discussion

The primary outcome of our study was that purslane supplementation decreased SBP (−3⋅64 mmHg), although it did not show a significant effect on DBP. In addition, it was found that there were significant decreases in weight (−0⋅73 kg) and BMI (−0⋅35 kg/m^2^). The secondary outcomes represented in the subgroup analysis were significant decreases in SBP, weight, and BMI in participants above 40 years old and under 30 kg/m^2^. Furthermore, a decrease in WC was shown after 8 weeks of supplementation and a BMI under 30 kg/m^2^. More specifically of data reported on this study, the supplementation consisted of purslane extract and seed, with daily dosages of between 180 mg extract and 10 g powder for a period of 5–12 weeks being advised. The included participants in this study were overweight and obese. Whereas the level of reducing BMI and weight is statistically significant, this is not clinically significant. In addition, it is important to find that purslane leads to a decreasing BMI. Following a 3-month follow-up, significant blood pressure reductions were defined as a 10 mmHg reduction in SBP or a 5 mmHg reduction in DBP. A 5 % weight loss after one year of treatment could be considered clinically significant weight loss.^(34)^

Systemic inflammation, impaired glucose tolerance, hypertension, and impaired glucose tolerance are all associated with obesity, each of which independently raises the risk of diabetes and CVD.^(35)^ Besides, hypertension is a major cause of CVD, which leads to premature death.^(36)^ It is important to consider the various doses utilised, variations in sample sizes, and length of the intervention to explain the contentious results shown across the research. According to research conducted on humans, consuming purslane seeds for 5 weeks may help those with T2DM have better anthropometric measurements, serum lipid levels, and blood pressure.^(23)^ Additionally, the findings of a different study demonstrated that consuming 60 mg of purslane significantly decreased SBP in the cohort as a whole.^(26)^ Contrary, The anthropometric indices (BMI, waist, and weight), as well as the serum levels of low-density lipoprotein, total cholesterol, and fasting blood sugar, were found to drop in women with MetS who consumed purslane for 12 weeks, although SBP and DBP were not significantly affected^(25)^ it is inconsistent that how decreasing anthropometric items not effect on the blood pressure which needs several studies on this area. Additionally, the results of a study by Darvish Damavandi *et al.* showed that a 12-week purslane supplement of 300 mg per day had no discernible impact on weight, WC, SBP, or DBP.^(28)^ Although Ghorbanian *et al.* showed that 1,200 mg/d purslane supplementation decreases BMI in overweight and obese participants.^(24)^ Aliniya *et al.* in NAFLD participants illustrated that 1 g/d purslane for 12 weeks had no effect on body fat percentage, waist-to-hip ratio, and BMI.^(37)^ Furthermore, a study that was performed on 3 g/d purslane leaves consumption with dinner showed potentially altered blood lipid metabolism and hypercholesterolaemia and led to a decreasing risk of heart disease^(38)^ as well and a meta-analysis showed the same result of supplementation higher than 1⋅5 g/d purslane lipid profile and glucose.^(39)^

The mechanisms through which purslane intake might affect weight loss and blood pressure have been unknown. Multiple mechanisms could account for its positive effects. The purslane seed's impact on insulin resistance may be responsible for weight loss.^(19)^ According to some documents, the noradrenaline concentration of purslane is what gives it its lipolytic properties.^(40)^ Due to its thiamine content, purslane may be more effective for obese, MetS, and diabetic patients. It has been reported that patients with obesity and diabetes often have low levels of thiamine, a coenzyme that converts carbohydrates, fats, and proteins into energy.^(19,41,42)^ Consequently, purslane consumption may help alleviate this thiamine deficit. Purslane's antihyperglycaemic effect may be attributed to its ability to increase insulin secretion by affecting membrane depolarisation and Ca^2+^ entry and closing the K^+^/ATP channels.^(43)^ Another study showed that eating purslane seeds could considerably raise T2DM patients’ glucagon-like peptide-1 levels.^(41,44)^ Purslane, which contains a high amount of flavonoids, might beneficially decrease CVD including high blood pressure.^(45)^ Omega-3 fatty acids have been demonstrated to have antihypertensive effects through a number of pathways, including an increase in endothelial nitric oxide production and a decrease in the activity of angiotensin-converting enzyme.^(46)^ Omega-3 unsaturated fats have a more defined effect on lowering SBP than DBP.^(46,47)^ Alpha lipoic acid administration might dramatically reduce SBP increase in two animal models of hypertension by inhibiting SIRT3 (sirtuin 3) decrease, superoxide dismutase 2 (SOD2) hyperacetylation, and mitochondrial reactive oxygen species (ROS) overproduction.^(48,49)^ Furthermore, purslane has been shown to have a positive impact on insulin resistance,^(50)^ potentially improving weight management.^(51)^ Additionally, purslane stimulates amp-activated protein kinase (AMPK) phosphorylation in adipocytes.^(52)^ The activity of AMPK regulates energy homeostasis in the cell. By stimulating browning, energy expenditure, glucose uptake, adiponectin secretion, and fatty acid oxidation, it simultaneously suppresses lipogenesis, lipolysis, and pro-inflammatory markers in adipose tissue.^(53,54)^ As a result, purslane activates AMPK to protect against obesity and metabolic dysfunction associated with it.^(55,56)^ In addition, purslane supplementation has been associated with a reduction in the growth and differentiation of adipocytes, as well as an increase in lipolysis and fatty acid oxidation,^(57)^ both known to suppress adipocytes. Purslane could also increase hepatic cholesterol 7a hydroxylase (CYP7A1) expression and, as a result, bile acids production.^(58)^ Bile acids appear to improve various MetS-related parameters.^(59)^ Bioactive components in purslane, including carotenoids, flavonoids, saponins, omega 3 s, and thiamine, may contribute to its anti-obesity effects.^(60)^ Using purslane homoisoflavonoids to stimulate 3 T3-L1 preadipocytes reduced lipid accumulation and downregulated adipogenic transcription factors, such as PPAR-c and CCAAT/enhancer-binding proteins.^(61)^ In the meantime, more studies are needed to reveal the other mechanisms involved in the anti-obesity effects of purslane. The fact that this is the first systematic review and meta-analysis to evaluate the effects of purslane supplementation on anthropometric measurements and blood pressure is one of its strengths, to the best of our knowledge. There are restrictions on our work that need to be made clear. The key drawbacks include small sample numbers, considerable heterogeneity for some variables, and the fact that purslane is primarily found in Iran. The impact of several confounding factors, including nutritional consumption, physical activity, drug misuse, alcohol use, and other relevant confounders, were not often considered in most research. Also, since different diseases were pooled, the results should be interpreted with caution. Furthermore, due to the lack of studies on normal-weight patients, perhaps this study finding does not give a good comparison in all groups’ patients.

## Conclusion

Existing evidence from RCTs in this meta-analysis suggests. Supplementing with purslane considerably lowered body weight, BMI, and SBP; however, WC and DBP did not see a decreasing effect. To confirm the effect of purslane supplementation on anthropometric measurements and blood pressure, more research is required.

## Supporting information

Narimani et al. supplementary material 1 Narimani et al. supplementary material

Narimani et al. supplementary material 2 Narimani et al. supplementary material

## Supplementary material

The supplementary material for this article can be found at https://doi.org/10.1017/jns.2023.115
