# Supplementary material for: The effects of purslane consumption on blood pressure, body weight, body mass index, and waist circumference: a systematic review and meta-analysis of randomised controlled
Source: J Nutr Sci. 2023 Dec 27;12:e129. doi: 10.1017/jns.2023.115 (PMC10753486; doi:10.1017/jns.2023.115)
Supplement: Narimani et al. supplementary material 1 — Narimani et al. supplementary material [file S2048679023001155sup001.docx]

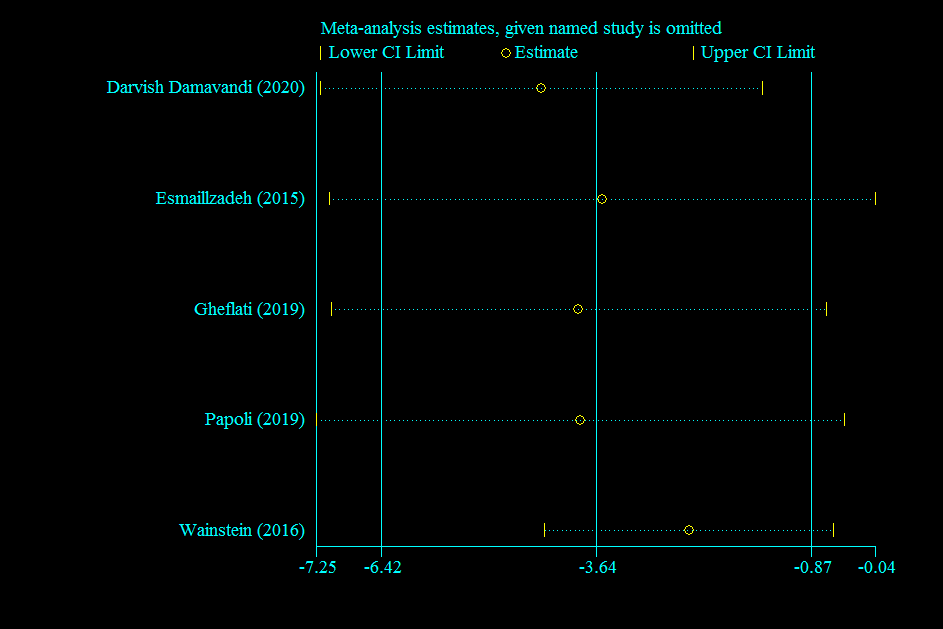


**Sup. Figure 1.** Influence analysis of purslane on SBP.

**
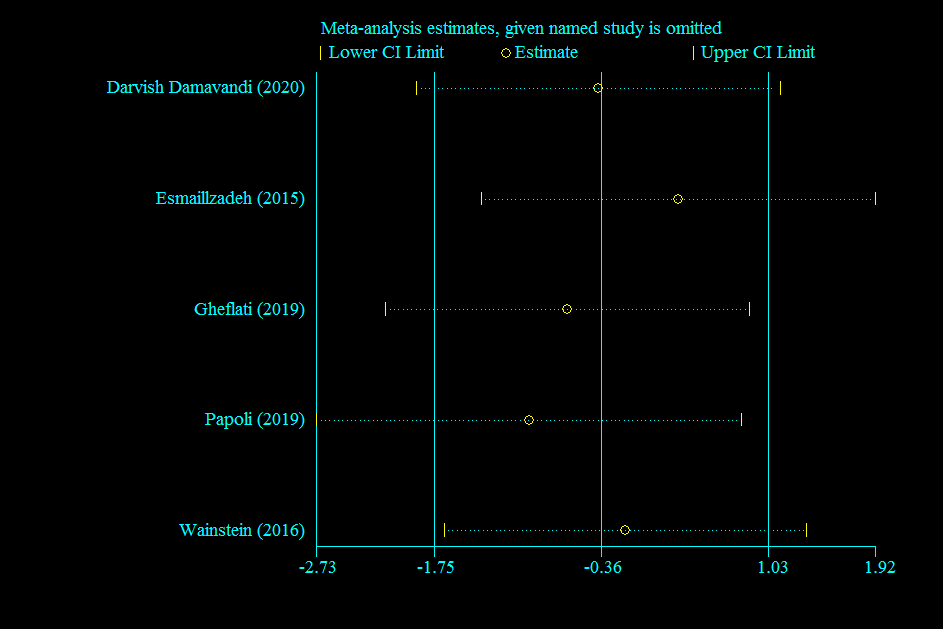
**

**Sup. Figure 2.** Influence analysis of purslane on DBP.

**
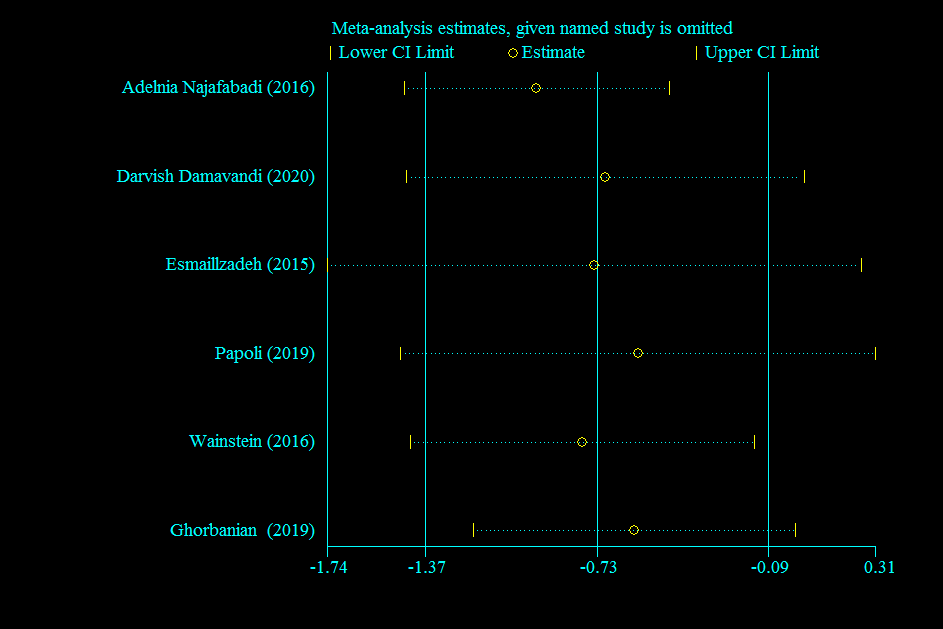
**

**Sup. Figure 3.** Influence analysis of purslane on weight.

**
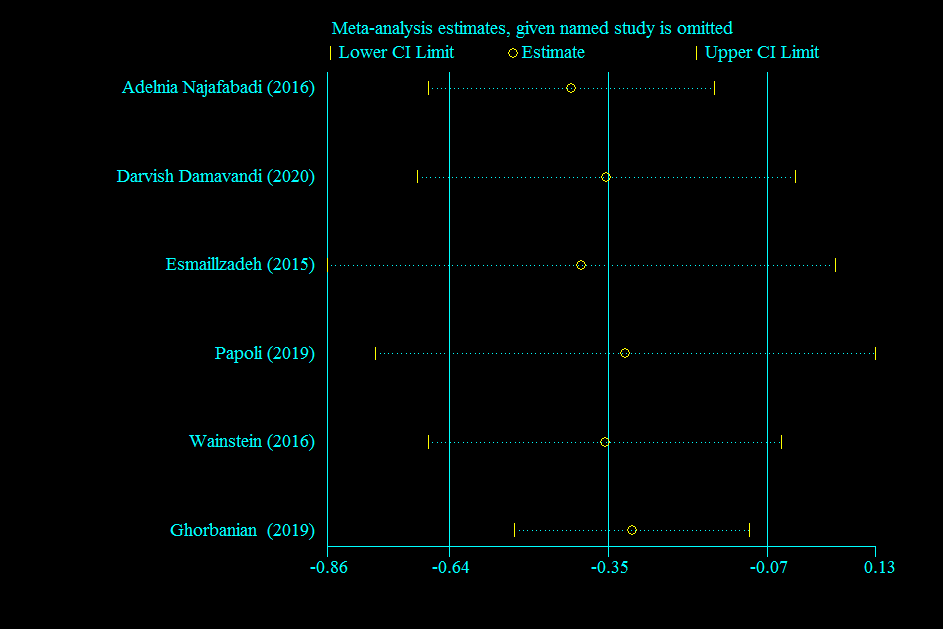
**

**Sup. Figure 4.** Influence analysis of purslane on BMI.

**
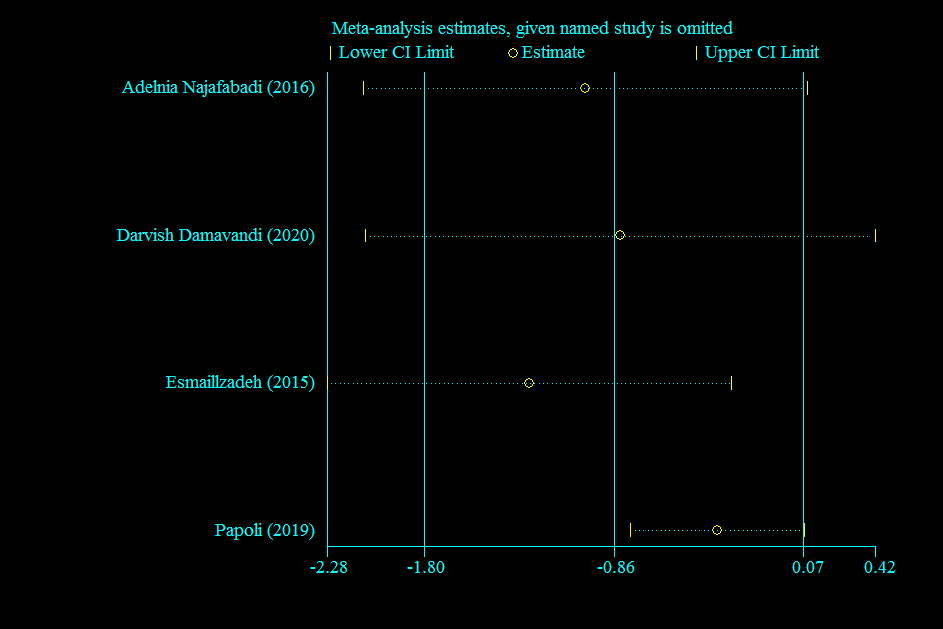
**

**Sup. Figure 5.** Influence analysis of purslane on WC.
